# Perioscope-Based Endoscopy in Modern Periodontal Care: A State-of-the-Art (SotA) Review

**DOI:** 10.7759/cureus.102753

**Published:** 2026-01-31

**Authors:** Vishnu Venu, Shankar S Menon, Arun Kurumathur Vasudevan

**Affiliations:** 1 Periodontics, Amrita School of Dentistry, Amrita Vishwa Vidyapeetham, Kochi, IND

**Keywords:** minimally invasive periodontal surgery, optical enhancement, periodontal endoscopy, perioscope, subgingival calculus

## Abstract

Periodontal therapy has traditionally relied on tactile sensation and indirect clinical indicators to guide subgingival instrumentation, a limitation that becomes increasingly evident in deep periodontal pockets and anatomically complex sites. Despite decades of refinement in nonsurgical periodontal therapy, residual subgingival calculus remains a major determinant of persistent inflammation and disease recurrence. Optical enhancement technologies have emerged to address this fundamental limitation, among which Perioscope-based periodontal endoscopy represents the most advanced clinically available modality enabling real-time visualization of the subgingival environment. This state-of-the-art (SotA) review critically synthesizes the current scientific and clinical evidence on Perioscope-assisted periodontal therapy, positioning it within the evolving paradigm of minimally invasive, precision-guided periodontics. Contemporary evidence demonstrates that Perioscope-assisted therapy significantly enhances detection and removal of subgingival calculus compared with conventional tactile methods. Optical magnification allows direct visualization of residual deposits, root surface morphology, and furcation anatomy, thereby redefining the procedural endpoint of nonsurgical debridement. Clinical studies consistently report superior reductions in bleeding on probing and gingival inflammation, while probing depth and clinical attachment outcomes appear comparable to conventional scaling and root planing in short-term evaluations. Beyond nonsurgical therapy, periodontal endoscopy has enabled the development of videoscope-assisted minimally invasive periodontal surgery, offering improved clinical outcomes with reduced morbidity, gingival recession, and patient discomfort. Emerging applications in peri-implant disease management further expand the scope of optical enhancement in periodontal care. In summary, Perioscope-based subgingival endoscopy represents the current pinnacle of optical enhancement in periodontal therapy. Its selective application in complex periodontal sites aligns with contemporary minimally invasive principles and signals a shift toward visually guided periodontal treatment. Continued technological refinement and long-term clinical trials will determine its future integration into standard periodontal practice.

## Introduction and background

Periodontal therapy has historically evolved through incremental refinements in mechanical debridement, instrumentation design, and adjunctive pharmacologic strategies. Despite these advances, the fundamental biological objective of periodontal treatment, which is the effective removal of subgingival biofilm and calculus, has remained constrained by a persistent technical limitation: the inability to directly visualize the subgingival environment during nonsurgical therapy [1-3]. This limitation becomes clinically consequential in deep periodontal pockets, multirooted teeth, and furcation-involved sites, where residual calculus is strongly associated with disease persistence and progression [4-6].

Dental calculus is not merely a secondary by-product of plaque mineralization: it is a biologically active substrate that harbors viable microorganisms and perpetuates inflammation through its porous structure and intimate attachment to diseased root surfaces [7-9]. Extensive histologic, extraction, and clinical studies have demonstrated that conventional scaling and root planing (SRP), even when performed by experienced clinicians, frequently leaves residual calculus in anatomically complex sites [10-12]. Surgical flap procedures improve access and visualization but introduce morbidity, gingival recession, and patient reluctance, creating a clinical trade-off between efficacy and invasiveness [13,14].

The past two decades have witnessed a paradigm shift toward minimally invasive and precision-guided periodontal therapy. Within this context, optical enhancement technologies have emerged as a logical solution to the visualization deficit inherent in conventional nonsurgical therapy. Periodontal endoscopy, specifically the Perioscope system, represents the most mature and clinically implemented optical modality enabling real-time visualization of the subgingival root surface under intact gingival tissues [15].

Unlike earlier magnification aids such as loupes or operating microscopes, which improve extraoral visualization only, periodontal endoscopy extends optical access directly into the periodontal pocket. This innovation has redefined nonsurgical periodontal therapy by converting it from a tactilely guided procedure into a visually verified intervention. As such, Perioscope-based endoscopy occupies a unique position at the intersection of micro-dentistry, minimally invasive therapy, and precision periodontics [16].

Recent advancements in optical technologies have further propelled the integration of digital enhancements into periodontal endoscopy, such as fluorescence-guided calculus detection and artificial intelligence-assisted image analysis, which promise to improve diagnostic accuracy and reduce operator variability [17]. These innovations build upon the foundational Perioscope system by incorporating real-time data processing to differentiate between healthy and diseased tissues more precisely, potentially minimizing overtreatment and enhancing patient-specific therapeutic strategies [18]. Moreover, the application of periodontal endoscopy has expanded beyond traditional nonsurgical debridement to include peri-implant disease management, where visualization of implant surfaces aids in targeted removal of biofilms without compromising implant integrity. This evolution underscores the shift toward multimodal, technology-driven approaches in periodontics, aligning with broader trends in precision medicine and minimally invasive interventions [15-17]. This state-of-the-art (SotA) review critically evaluates how Perioscope-based subgingival endoscopy has reshaped contemporary periodontal therapy, emphasizing technological evolution, current clinical evidence, unresolved controversies, and future trajectories.

## Review

Methodology

This review was conducted as a SotA narrative synthesis with the objective of critically integrating the most influential, methodologically sound, and clinically relevant evidence defining the current ceiling of Perioscope-based periodontal endoscopy. A SotA framework was selected to allow comprehensive integration of clinical outcomes, biological relevance, and technological evolution, particularly in a field characterized by methodological heterogeneity and rapidly advancing innovation, which limits the feasibility of quantitative meta-analytic synthesis. Accordingly, emphasis was placed not merely on summarizing outcomes, but on contextual interpretation, clinical applicability, and identification of unresolved controversies.

A targeted electronic literature search was performed using PubMed/MEDLINE, Scopus, and Web of Science databases for studies published up to late 2025. Search terms included combinations of “periodontal endoscopy”, “perioscope”, “subgingival endoscopy”, “endoscopy-assisted scaling and root planing”, “videoscope-assisted minimally invasive periodontal surgery”, “subgingival calculus detection”, and “optical enhancement in periodontics”. In addition, the reference lists of relevant systematic reviews and primary studies were manually screened to identify further pertinent publications. Priority was given to randomized controlled trials, prospective and retrospective clinical studies, extraction studies evaluating residual calculus, systematic reviews, meta-analyses, and key technological reports directly relevant to perioscope-based therapy.

Eligible studies included peer-reviewed publications evaluating the clinical, biological, or technological aspects of periodontal endoscopy in nonsurgical therapy, minimally invasive periodontal surgery, or peri-implant disease management. Studies limited exclusively to conventional scaling and root planing without optical or endoscopic assistance, non-peer-reviewed sources, isolated case reports, and publications lacking clinical or mechanistic relevance were excluded. Rather than cataloguing all available literature, study selection prioritized publications that advanced conceptual understanding, represented technological milestones,or clarified clinical efficacy and limitations.

Data were extracted descriptively, focusing on calculus detection accuracy, residual calculus removal, bleeding on probing and inflammatory outcomes, probing pocket depth and clinical attachment level changes, applications in minimally invasive periodontal surgery, and emerging peri-implant indications. Given the heterogeneity of study designs and outcome measures, formal quantitative risk-of-bias assessment tools were not applied. Instead, methodological quality, including study design, presence of control groups, objective outcome assessment, and extraction-based validation, was qualitatively considered during evidence weighting and interpretation. Evidence synthesis was performed narratively to highlight areas of consensus, clinical relevance, technological evolution, and unresolved limitations. Where available, findings from contemporary systematic reviews and meta-analyses were incorporated to provide quantitative context and enhance interpretive robustness [1-3].

Results

Enhanced Subgingival Visualization and Calculus Detection

The contemporary literature consistently identifies enhanced visualization and improved calculus detection as the principal advantages of Perioscope-based periodontal endoscopy. Comparative clinical studies demonstrate that endoscopic visualization significantly outperforms tactile exploration in identifying subgingival calculus, particularly in furcation-involved, interproximal, and deep periodontal sites, where tactile discrimination is inherently limited [17,18]. These findings underscore the limitations of conventional tactile assessment and highlight the diagnostic advantage conferred by direct optical visualization of the subgingival root surface.

Residual Calculus Removal: Evidence from Extraction and Clinical Studies

Extraction-based investigations provide objective validation of these observations by enabling post-treatment assessment of residual calculus. Teeth treated using endoscope-assisted instrumentation exhibit significantly lower residual calculus scores compared with those treated using conventional scaling and root planing, with the greatest benefit observed in multirooted teeth and sites with probing depths of 6 mm or greater [19,20]. These studies confirm that the superiority of periodontal endoscopy reflects a true mechanical advantage rather than subjective operator perception.

Inflammatory Outcomes: Bleeding on Probing and Gingival Health

Clinical outcome studies consistently report superior reductions in bleeding on probing and gingival inflammation following Perioscope-assisted therapy compared with conventional nonsurgical periodontal treatment, indicating improved inflammatory resolution [21,22]. Given the established role of bleeding on probing as a surrogate marker of periodontal inflammation, these improvements suggest that enhanced calculus removal achieved under endoscopic guidance translates into a more favorable subgingival biological environment [21-23].

Structural Outcomes: Probing Depth and Clinical Attachment Level

However, improvements in probing pocket depth and clinical attachment level are less consistently superior when compared with conventional therapy. Most randomized clinical trials report comparable reductions in probing depth and gains in clinical attachment level between treatment modalities during short-term follow-up periods [23,24]. Meta-analytic data indicate modest mean differences with overlapping 95% confidence intervals, suggesting limited overall superiority of endoscopy-assisted therapy for structural outcomes [25].

Site-Specific and Anatomical Considerations

Recent systematic reviews and meta-analyses further corroborate these findings, reporting statistically significant reductions in residual calculus favoring endoscopy-assisted therapy, with pooled effect estimates demonstrating narrow 95% confidence intervals and low-to-moderate heterogeneity, reflecting variability in study design and operator experience [25,26]. Despite this, site-specific analyses reveal greater benefits of Perioscope-assisted therapy in deep periodontal pockets and anatomically complex regions, including furcation-involved and multirooted teeth, where conventional instrumentation is less effective [23-25]. These findings support the selective clinical advantage of periodontal endoscopy in challenging anatomical scenarios rather than its routine use across all periodontal sites.

Evidence from Systematic Reviews and Meta-Analyses

Systematic reviews and meta-analyses consistently acknowledge enhanced calculus removal as the strongest and most reproducible benefit of periodontal endoscopy while also noting increased treatment time and technique sensitivity as practical limitations [25,26]. Emerging evidence suggests modest radiographic and clinical advantages in deeper defects, although long-term data remain limited [26]. Collectively, the available evidence supports Perioscope-based periodontal endoscopy as a modality that significantly improves calculus detection and inflammatory control, with selective advantages in anatomically complex periodontal sites.

Discussion

Biological Rationale for Subgingival Visualization

The present SotA review seeks to reinforce Perioscope-based subgingival endoscopy as a pivotal technological advancement addressing a long-standing limitation in periodontal therapy: the inability to directly visualize the subgingival environment during nonsurgical treatment [1-3]. Despite decades of refinement in mechanical instrumentation and adjunctive approaches, nonsurgical periodontal therapy has remained fundamentally constrained by blind instrumentation, particularly in deep periodontal pockets, furcation-involved teeth, and anatomically complex root surfaces [8-12]. From a contemporary perspective, periodontal endoscopy represents not merely an adjunctive device, but a conceptual shift in how periodontal debridement is performed, verified, and evaluated within precision-guided care paradigms [15,16].

Clinical Significance of Improved Calculus Detection

Residual subgingival calculus has been unequivocally linked to persistent inflammation, delayed healing, and disease recurrence [4-6]. Histologic and extraction studies consistently demonstrate that calculus deposits frequently remain following conventional scaling and root planing, even when performed meticulously by experienced clinicians [9-12]. The porous structure of calculus, its intimate attachment to diseased root surfaces, and its capacity to harbor viable microorganisms render it a biologically active substrate rather than an inert by-product of plaque mineralization [7-9]. Consequently, incomplete calculus removal undermines the biological objectives of periodontal therapy regardless of operator skill or instrument selection [10,11].

Traditional scaling and root planing relies primarily on tactile sensation to detect residual deposits, a method that is inherently subjective and limited by root surface anatomy, operator experience, and access constraints [12,13]. Numerous investigations have demonstrated that tactile explorers lack adequate sensitivity and specificity for subgingival calculus detection, particularly in deep pockets and furcation regions [12,13]. Within this biological and technical context, continued reliance on tactile smoothness as the procedural endpoint appears increasingly incongruent with contemporary precision-based periodontal care [14,15].

Periodontal endoscopy directly addresses this mismatch by enabling real-time visualization of the subgingival root surface under intact gingival tissues [15,16]. This capability redefines the endpoint of nonsurgical periodontal therapy from a subjective tactile impression to an objectively verified visual outcome [17]. From a SotA standpoint, this shift aligns periodontal therapy with other surgical disciplines in which endoscopic visualization has long been considered indispensable for procedural accuracy and predictability [16,18].

Among all evaluated outcome domains, enhanced calculus detection and removal constitute the most robust and reproducible evidence supporting periodontal endoscopy [17-20]. Comparative clinical studies consistently demonstrate that endoscopic visualization significantly outperforms tactile exploration in identifying subgingival calculus, particularly in furcation-involved and interproximal sites where tactile discrimination is inherently limited [17,18]. Extraction-based studies provide especially compelling validation, as they permit objective post-treatment assessment of residual calculus [19,20]. Teeth treated with endoscope-assisted debridement exhibit significantly lower residual calculus scores compared with those treated using conventional scaling and root planing, with the greatest benefit observed in multirooted teeth and pockets ≥6 mm [19,20].

Recent systematic reviews and meta-analyses further strengthen this evidence base by demonstrating statistically significant reductions in residual calculus favoring periodontal endoscopy, with reported confidence intervals consistently supporting this effect despite moderate heterogeneity across studies [25,26]. These findings confirm that the benefits of endoscopic visualization represent a genuine mechanical advantage rather than a perceived improvement driven by operator bias or enthusiasm [18-20]. Importantly, no other currently available nonsurgical adjunct directly addresses the visualization deficit inherent in conventional scaling and root planing [6,14].

Inflammatory Resolution Versus Structural Healing

Clinical studies consistently report superior reductions in bleeding on probing and gingival inflammation following Perioscope-assisted therapy compared with conventional nonsurgical treatment [21,22]. Given the established role of bleeding on probing as a surrogate marker for periodontal inflammation and disease activity, these improvements are clinically meaningful and suggest more effective inflammatory control [21-23]. Enhanced visualization and improved calculus removal likely contribute to the establishment of a more favorable subgingival biological environment [22,23].

However, improvements in probing pocket depth and clinical attachment level are less consistently superior when compared with conventional therapy [23-25]. In many randomized controlled trials, probing depth reduction and attachment gains following Perioscope-assisted therapy are comparable rather than significantly greater [23,24]. Meta-analytic data similarly demonstrate modest mean differences with overlapping confidence intervals [25]. This absence of universal superiority in structural outcomes has been cited as a criticism of periodontal endoscopy; however, such interpretation overlooks the multifactorial biological determinants of attachment gain, including defect morphology, host response, systemic health, smoking status, and patient compliance [26,27]. From a SotA analytical perspective, these findings underscore that periodontal endoscopy functions primarily as a precision-enhancing modality rather than a regenerative intervention [15,24].

Notably, site-specific analyses reveal greater benefits in deep pockets and anatomically complex regions, including multirooted posterior teeth and narrow interproximal spaces [23-25]. These observations support a selective, indication-driven application of periodontal endoscopy rather than indiscriminate use across all periodontal sites [16,27].

Implications for Nonsurgical Periodontal Therapy

Periodontal endoscopy challenges the traditional dichotomy between nonsurgical and surgical periodontal therapy [14-16]. Historically, improved visualization was achievable only through surgical flap elevation, at the cost of increased morbidity, gingival recession, and patient discomfort [13,14]. Endoscopic visualization disrupts this paradigm by providing surgical-level visualization without surgical access [15-17]. This capability refines decision-making by allowing clinicians to more accurately assess nonsurgical treatment endpoints and determine when surgical intervention is genuinely indicated [15,16].

Applications in Minimally Invasive Periodontal Surgery

The extension of endoscopic visualization into videoscope-assisted minimally invasive periodontal surgery represents a logical evolution of optical enhancement technology [28,29]. Clinical studies demonstrate favorable outcomes, including significant probing depth reduction, clinical attachment gain, reduced gingival recession, and improved patient-reported outcomes compared with conventional open-flap procedures [28,29]. These findings illustrate how optical enhancement may reconcile the competing demands of surgical access and tissue preservation within contemporary periodontal practice [28].

Emerging Applications in Peri-Implant Disease

Peri-implant diseases present unique diagnostic and therapeutic challenges due to differences in tissue attachment and implant surface microtopography [30]. Conventional mechanical debridement techniques often struggle to achieve adequate cleaning without damaging implant surfaces [30]. Emerging evidence suggests that endoscopic visualization may enhance precision during peri-implant debridement by enabling direct inspection and targeted deposit removal [30]. While this application remains in its early stages, it represents a promising extension of optical enhancement beyond natural dentition.

Limitations, Barriers to Adoption, and Controversies

Despite its demonstrated advantages, periodontal endoscopy has not achieved widespread adoption, and several unresolved challenges and controversies persist [24-26]. A steep learning curve remains a significant barrier, as effective use requires advanced hand-eye coordination and interpretation of indirect visual feedback [24]. Operator-dependent variability contributes to inconsistent outcomes across studies, particularly during early adoption phases [25]. Increased treatment time, equipment costs, and the need for specialized training raise legitimate concerns regarding clinical efficiency, cost-effectiveness, and accessibility [24,25]. Furthermore, the lack of consistent superiority in probing depth and attachment outcomes has fueled debate regarding its routine use versus selective application [23-25].

The current evidence base is also limited by relatively short follow-up periods and methodological heterogeneity, which restrict definitive conclusions regarding long-term periodontal stability and tooth survival [26]. These criticisms highlight the need for standardized protocols, long-term randomized trials, and economic evaluations to more precisely define the role of periodontal endoscopy within routine care.

Future Directions and Digital Integration

Future advances in periodontal endoscopy are closely tied to integration with digital technologies [16,27]. Fluorescence-guided calculus detection, digital image processing, and artificial intelligence-assisted image interpretation represent promising avenues for reducing operator dependence and improving reproducibility [16,27]. Specific research questions warranting investigation include whether AI-driven calculus recognition algorithms can standardize detection accuracy, whether fluorescence-based systems improve inter-operator consistency, and whether real-time feedback mechanisms can shorten learning curves and reduce treatment time. Additional areas of inquiry include evaluating the diagnostic accuracy of digital endoscopic analytics, the role of automated documentation in longitudinal disease monitoring, and the cost-effectiveness of digitally enhanced endoscopic workflows.

Viewed through a SotA lens, Perioscope-based periodontal endoscopy represents a convergence of biological insight, technological innovation, and minimally invasive philosophy [15-17]. Its greatest contribution lies in redefining nonsurgical periodontal therapy by introducing visual verification as the standard for mechanical debridement [16-18]. While it is not a universal replacement for conventional therapy, periodontal endoscopy exemplifies the transition toward precision-guided, patient-centered periodontal care [27-29]. Continued technological refinement and well-designed longitudinal studies will ultimately determine its integration into routine periodontal practice.

## Conclusions

Perioscope-based periodontal endoscopy represents a transformative optical enhancement in periodontal therapy, enabling direct visualization of the subgingival environment during non-surgical treatment. The available evidence indicates superior calculus detection and removal, improved inflammatory outcomes, and enhanced precision in anatomically complex sites. While probing depth and attachment level gains are comparable to conventional SRP, the reduced reliance on surgical access and improved treatment control highlight its clinical value. Recent advancements, including AI-assisted image analysis and fluorescence-guided detection, further amplify its potential by automating calculus identification and minimizing procedural variability, paving the way for broader clinical adoption. Longitudinal data from studies conducted up to 2025 suggest sustained improvements in periodontal stability and reduced recurrence rates, particularly in patients with severe periodontitis, underscoring its role in long-term disease management. As the field progresses toward integrated digital ecosystems, Perioscope-based endoscopy will likely become integral to personalized, minimally invasive protocols, bridging non-surgical and regenerative therapies for optimal patient outcomes. Further long-term, well-designed randomized trials are required to establish standardized protocols and define its role as a routine adjunct in periodontal care.
